# Side of Lesions Predicts Surgical Outcomes in Patients With Drug-Resistant Temporal Lobe Epilepsy Secondary to Focal Cortical Dysplasia Type IIIa

**DOI:** 10.3389/fneur.2020.580221

**Published:** 2020-12-10

**Authors:** Xinghui He, Dingyang Liu, Zhuanyi Yang, Junmei Zhang, Sushan Li, Zhiquan Yang

**Affiliations:** Department of Neurosurgery, Xiangya Hospital, Central South University, Changsha, China

**Keywords:** drug-resistant epilepsy, temporal lobe epilepsy, focal cortical dysplasia type IIIa, anterior temporal lobectomy, seizure outcomes

## Abstract

**Objective:** This study aims to evaluate the surgical outcomes and analyze the predictors of surgical outcomes in patients undergoing anterior temporal lobectomy (ATL) for drug-resistant temporal lobe epilepsy (TLE) secondary to focal cortical dysplasia (FCD) type IIIa.

**Methods:** Data on patients with drug-resistant TLE secondary to FCD type IIIa who had undergone ATL at Xiangya Hospital, Central South University from January 2014 to April 2018, were collected retrospectively. International League Against Epilepsy (ILAE) classification was used to evaluate postoperative seizure outcomes. Predictors of surgical outcomes were identified by using univariate and multivariate analyses.

**Results:** A total of 43 patients with drug-resistant TLE secondary to FCD type IIIa who had undergone ATL were included in this study. Twenty patients had right ATL, and 23 patients had left ATL. With a follow-up of 2–6 years, 76.7% (33 of 43) of patients were seizure-free. Univariate and multivariate analysis results indicated that lesions on the right side independently predict postoperative seizure freedom (OR, 0.08; 95% CI, 0.01–0.72; *P* = 0.024).

**Conclusion:** ATL is an effective therapy for patients with drug-resistant TLE secondary to FCD type IIIa. Patients with lesions on the right side are more likely to achieve postoperative seizure freedom.

## Introduction

Temporal lobe epilepsy (TLE) is considered as the most common type of epilepsy that is resistant to antiepileptic drugs (AEDs) (1). For carefully selected patients with drug-resistant TLE, resection surgery is an effective therapy option because seizures cannot be wholly controlled in these patients after taking two or more well-tolerated and properly selected AEDs (2–5). In patients with drug-resistant TLE, the most common neuropathological diagnosis is hippocampal sclerosis (HS) that is coexisted or not with other pathologies such as focal cortical dysplasia (FCD), vascular malformations, tumors, etc. (6).

When hippocampal sclerosis (HS) and FCD type I in the adjacent temporal lobe are coexisting, FCD type IIIa is diagnosed (7). It is hypothesized that the HS in FCD type IIIa is a neuropathological change caused by the seizures triggered by the FCD in the temporal lobe (7). However, there are no substantial differences in semiology and electrophysiology in patients with HS alone and FCD type IIIa (8–10). Recently, FCD type IIIa is reported as a factor that may influence the seizure outcomes in patients with TLE who have undergone anterior temporal lobectomy (ATL) (8, 11, 12). However, the results are contradictory. Some studies have reported that patients with FCD type IIIa had similar surgical outcomes compared to patients with isolated HS (10, 12), and Johnson et al. (8) have reported that patients with FCD type IIIa had poorer surgical outcomes compared to patients with isolated HS, while Dührse et al. (11) have found that patients with FCD type IIIa had significantly better seizure outcomes than patients with isolated HS. Besides, no study to date has evaluated the predictive factors of surgical outcome in patients with drug-resistant TLE associated with FCD type IIIa.

Herein, to evaluate the postoperative seizure outcomes and analyze the predictive factors of surgical outcome in patients with drug-resistant TLE associated with FCD type IIIa, the present study reports a case series containing 43 cases with drug-resistant TLE secondary to FCD type IIIa who have undergone anterior temporal lobectomy.

## Methods

### Patients Selection

This study was approved by the ethics committee of Xiangya Hospital, Central South University. Written informed from all patients, or their guardians' consent was obtained.

Data from cases with drug-resistant TLE secondary to FCD IIIa who had undergone ATL at Xiangya Hospital Central South University from January 2014 to April 2018 were collected.

The exclusion criteria were: (1) patients with extratemporal lobe epilepsy; (2) patients with neuropathology other than FCD type IIIa; (3) patients with structural lesions such as glial scar, tumors, arteriovenous malformation, etc. in the temporal lobe; (4) patients who had undergone reoperations due to epilepsy; (5) patients who had undergone corpus callosotomy, and vagus nerve stimulation, etc.

The inclusion criteria were: (1) patients who had undergone ATL for drug-resistant TLE; (2) the histopathological diagnosis revealed that HS coexists with FCD type I in the adjacent temporal lobe; (3) patients who had a follow-up of ≥2 years after surgery.

Demographic and clinical information of patients who have met the inclusion criteria were collected from patients' medical records retrospectively. The collected demographic and clinical information included sex, age at surgery, age at seizure onset, seizure duration, seizure frequency, seizure types, past medical history, side of lesions, MRI results, results of 18F-FDG-PET, results of EEG, the performance of invasive EEG, and surgical complications.

### Preoperative Evaluation

All patients have undergone the evaluation of medical history, neurological examination, seizure semiology, long-term video scalp electroencephalography (EEG), and 3.0 Tesla high-resolution brain magnetic resonance imaging (MRI) with T1, T2, and fluid-attenuated inversion recovery (FLAIR) sequences as standard investigations. If needed, additional examinations such as invasive EEG and/or 18F-fluorodeoxyglucose positron emission tomography (18F-FDG-PET) were also used to help to locate the epileptogenic zones. After completion of these presurgical evaluations, surgical decisions were made based on the preoperative discussions among neurosurgeons, neurologists, neuroradiologists, and electrophysiologists.

### Surgical Procedure

All patients had undergone a standard ATL. In the right hemisphere, the extent of resection limited to 4.0–5.0 cm of the anterolateral temporal lobe. The extent of resection limited to 3.0–4.0 cm of the anterolateral temporal lobe in the left hemisphere because of the neighborhood of eloquent speech centers. The resection of the mesial structures in both hemispheres included resection of the anterior 3.0 cm of the hippocampus and the amygdala. The resected anterior lateral temporal lobe and hippocampus were sent for histological evaluation separately.

### Follow Up and Seizure Outcomes

Patients were followed up for 2–6 years. The International League Against Epilepsy (ILAE) classification (13) during the last 2 years of follow-up was used to classify the postoperative seizure outcomes. For patients in ILAE classes 1 and 2, favorable outcomes were defined. For patients in ILAE classes 3–6, unfavorable outcomes were defined.

### Statistical Analysis

The SPSS software (version 18.0, USA) was used for statistical analysis, and a *p*-value < 0.05 was considered statistically significant. Youden's index in a receiver operating characteristic curve analysis was used to detect the cutoff values of continuous variables that may influence surgical outcomes. Univariate analysis was performed using Pearson's chi-square test or Fisher's exact test. Variables showing a *P*-value < 0.2 in the univariate analysis were selected as candidates for multivariate analysis, and the Cramer's V was used to test the intercorrelations between these variables first. After ruling out high intercorrelations between covariates (Cramer's V < 0.5),these variables were recruited into a binary logistic regression model in a backward manner.

## Results

### Patient Characteristics

A total of 43 patients who had undergone ATL for drug-resistant TLE associated with FCD type IIIa were included, and all patients were right-handed in the present study. Twenty-six (60.5%) were men and 17 (39.5%) were women. The mean ± SD age at surgery was 27.3 ± 9.4 years (range: 11.0–59.0 years); the mean ± SD duration of seizures (the time spans between the age at seizure onset and age at surgery) was 13.7 ± 8.1 years (range: 1.0–30.0 years); and the mean ± SD age at seizure onset was 13.5 ± 8.3 years (range 0.0–30.5 years). Focal aware were reported in 17 (39.5%) patients. Twenty (46.5%) patients had focal seizures only, while the other 23 (53.5%) patients had focal to bilateral tonic-clonic seizures. Besides, one (2.3%) patient had experienced status epilepticus, and one (2.3%) patient had acute postoperative seizures. These two variables were not evaluated as predictors of surgical outcomes because of small sample sizes. The mean ± SD monthly seizure frequency was 11.7 ± 22.4 times. Epilepsy risk factors occurred in 9 (20.9%) patients. Five (11.6%) had a history of head trauma, three (7.0%) had a history of febrile seizures, and one (2.3%) had a history of craniotomy (resection of arachnoid cyst of middle cranial fossa).

### Findings of Preoperative Evaluation

The findings of brain MRI scans showed HS in all patients. Twenty (46.5%) patients had right HS, and 23 (53.5%) patients had left HS. Besides, temporal lobe atrophy ipsilateral to HS was observed in two (4.7%) patients.

A standard 10-20 system with a 64-channel electrode system was used to perform long-term video EEG monitoring. All patients received at least 24 h of long-term video EEG monitoring before surgery. During the EEG recording, the interictal epileptic discharges (IEDs) were observed in all patients. The IEDs of 21 (48.8%) patients were unilateral, and the IEDs of the other 22 (51.2%) patients were bilateral. Seizures were successfully recorded in 25 (58.1%) patients, 16 (37.2%) of whom had unilateral ictal onset rhythms (IOR), and 9 (20.9%) of whom had bilateral IOR. Monitored of the IOR was discontinued in 18 (41.9%) patients because of sparse seizure frequencies. For patients whom the IOR were not monitored, the surgical side was determined according to the evaluations of seizure semiology, the findings of preoperative MRI scan, the results of IEDs, and the results of FDG-PET (FDG-PET was performed in 17 of these 18 patients).

FDG-PET was performed in 36 (83.7%) patients, and regions of hypometabolism were observed. The regions of hypometabolism in 25 (58.1%) patients were located in the regions of the anterior temporal lobe, which were removed postoperatively. The remaining 11 (25.6%) patients had regions of hypometabolism beyond the anterior temporal lobe. In addition, 6 (14.0%) patients have received invasive EEG monitoring because the results of non-invasive evaluations were non-congruent.

### Seizure Outcomes and Neuropathology

Among the 43 patients, 20 (46.5%) patients had right ATL, and 23 (53.5%) patients had left ATL. With a mean ± SD follow-up of 3.2 ± 0.7 years, seizure freedom was observed in 33 (76.7%) patients [32 (74.4%) patients were in ILAE class 1 outcome, and 1 (2.3%) patient was in ILAE class 2 outcome]. Of the remaining 10 (23.3%) patients, 3 (7.0%) belonged to the ILAE class 3 outcome, 4 (9.3%) belonged to the ILAE class 4 outcome, and 3 (7.0%) belonged to ILAE class 5 outcome.

Surgical specimens were processed for routine histological analysis. HS and FCD type I in the adjacent temporal lobe were observed in all patients, and then FCD type IIIa was diagnosed.

### Surgical Complications

Surgical complications occurred in 4 (9.3%) patients. Intracranial bleeding occurred in 1 (2.3%) patient, subarachnoid hemorrhage was observed in 1 (2.3%) patient, and intracranial infection occurred in 1 (2.3%) patient, none of these patients needed surgical treatment. One (2.3%) patient had postoperative mania in which medical treatment was required to control the symptom of mania. Quadrantanopia was observed in 27 (62.8%) patients. However, it was not considered a surgical complication because of its expectative. No perioperative deaths occurred.

### Predictors of Seizure Outcomes

The predictors of postoperative seizure outcomes were analyzed using univariate and multivariate analyses. In the univariate analyses, side of lesions was significantly associated with seizure outcomes (*P* = 0.023), and three other variables including age at surgery (*P* = 0.07), age at seizure onset (*P* = 0.06), and the findings of IEDs (*P* = 0.085) showed a *P*-value of < 0.2 although the statistical differences were not significant (Table 1). After ruling out high intercorrelations between covariates (Cramer's V < 0.5; Supplementary Table 1), these four variables were recruited into the binary logistic regression model in a backward manner. The multivariate analysis results showed that the lesions on the right side were the only factor that independently predicts postoperative seizure freedom (Table 2).

**Table 1:** Patients' demographic and clinical characteristics and their relationship with seizure outcomes (*N* = 43), No. (%).

**Variables**	**Favorable**	**Unfavorable**	***P* value**
**Sex**			
Male	20 (76.9)	6 (23.1)	0.999^#^
Female	13 (76.5)	4 (23.5)	
**Age at surgery**			
≤ 27.5 years	16 (94.1)	1 (5.9)	0.070^#^
>27.5 years	17 (65.4)	9 (34.6)	
**Seizure duration**			
≤ 20 years	27 (77.1)	8 (22.9)	0.999^#^
>20 years	6 (75.0)	2 (25.0)	
**Age at seizure onset**			
≤ 16 years	23 (88.5)	3 (11.5)	0.060^#^
>16 years	10 (58.8)	7 (41.2)	
**Seizure frequency**			
Monthly	29 (80.6)	7 (19.4)	0.394^#^
Sparse	4 (57.1)	3 (42.9)	
**Seizure types**			
Focal	15 (75.0)	5 (25.0)	0.999^#^
focal to Bilateral tonic-clonic seizures	18 (78.3)	5 (21.7)	
**Auras**			
Yes	13 (76.5)	4 (23.5)	0.999^#^
No	20 (76.9)	6 (23.1)	
**Epileptic risk factors**			
Yes	8 (88.9)	1 (11.1)	0.599^#^
No	25 (73.5)	9 (26.5)	
**Side of lesions**			
Right	19 (95.0)	1 (5.0)	0.023^#^^*^
Left	14 (60.9)	9 (39.1)	
**MRI Result**			
HS only	32 (78.0)	9 (22.0)	0.415^##^
HS + TLS	1 (50.0)	1 (50.0)	
**Performance of 18F-FDG-PET**			
Yes	28 (77.8)	8 (22.2)	0.999^#^
No	5 (71.4)	2 (28.6)	
**Region of hypometabolism in 18F-FDG-PET**			
Anterior temporal lobe	20 (80.0)	5 (20.0)	0.863
Beyond the anterior temporal lobe	8 (72.7)	3 (27.3)	
Unknow	5 (71.4)	2 (28.6)	
**Results of IEDs**			
Unilateral	19 (90.5)	2 (9.5)	0.085^#^
Bilateral	14 (63.6)	8 (36.4)	
**Ictal onset rhythms**			
Unilateral	11 (68.8)	5 (31.3)	0.376
Bilateral	6 (66.7)	3 (33.3)	
Unknow	16 (88.9)	2 (11.1)	
**Invasive EEG**			
Yes	4 (66.7)	2 (33.3)	0.913^#^
No	29 (78.4)	8 (21.6)	
**Surgical complications**			
Yes	2 (50.0)	2 (50.0)	0.226^##^
No	31 (79.5)	8 (20.5)	

*sGTCS, secondary generalized tonic-clonic seizure; HS, hippocampal sclerosis; TLS, temporal lobe sclerosis; 18F-FDG-PET, 18F-fluorodeoxyglucose positron emission tomography; IEDs, interictal epileptic discharges; EEG, electroencephalography*.

# *For comparisons of binary variables, Chi square test with continuity correction was used*.

## *For comparisons of binary variables, the Fisher's exact was used*.

* *P < 0.05*.

**Table 2:** Predictors of surgical outcomes in patients with drug-resistant temporal lobe epilepsy secondary to focal cortical dysplasia type IIIa on multivariate analysis.

**Variables**	**OR**	**95% CI**	***P* value**
Age at surgery ≤ 27.5 years	0.21	0.02–2.04	0.177
Age at seizure onset ≤ 16 years	0.74	0.10–5.39	0.767
Side of lesions (right)	0.08	0.01–0.72	0.024^*^
IEDs (bilateral)	3.07	0.48–19.54	0.235

*OR, odds ratio; CI, confidence interval*.

* * P < 0.05*.

## Discussion

In 2011, the subtypes of FCD were redefined by the International League Against Epilepsy (ILAE). The coexistence of HS and FCD type I in the adjacent temporal lobe was classified as FCD type IIIa (7). It has been reported that more than 40% of patients with HS is associated with FCD type I in the temporal lobe (12, 14, 15). However, the surgical outcomes in patients who had undergone ATL for TLE associated with FCD type IIIa are still on a debate (8, 11), and little is known about the prognostic factors of surgical outcomes in such a patient cohort. In this study, we report a case series containing 43 patients who have undergone ATL for TLE associated with FCD type IIIa. To the best of our knowledge, none of the previous studies have analyzed the predictive factors of surgical outcomes in patients with TLE secondary to FCD type IIIa.

Many studies have reported the surgical outcomes in patients TLE associated with HS. In large series on surgical outcomes in patients TLE associated with HS, the seizure-free rate ranges from 49 to 78% (16–20). These differences in postoperative seizure outcomes may be attributable to the cases chosen, surgical types, the timing of seizure outcome evaluation, and the subtypes of neuropathology. In these studies, patients with TLE secondary to FCD type IIIa might also be included. However, FCD type IIIa was not classified as a type of pathology other than HS because of the research spans. Recently, some studies have reported the surgical outcomes in patients with TLE secondary to FCD type IIIa (8, 11, 12, 14). However, the postoperative seizure outcomes are heterogeneous because of the limited sample size, the rate of patients in Engle class I ranges from 41.7 to 84% (11, 12, 14). In this study, we observed that 76.7% of patients who had undergone ATL for TLE associated with FCD type IIIa were seizure-free with a mean follow-up of 3.2 years.

Knowledge of surgical safety is another crucial aspect for both patients and neurosurgeons during preoperative counseling and surgical decision-making. The rate of surgical complications in previous literature varies partly due to different postoperative evaluation standards and surgical techniques (21, 22). During the last decades, epilepsy surgery complication rates have dramatically decreased over time (21, 23). The rates of surgical complications in patients who underwent resection surgery for TLE are considered to be < 5.2%, and the permanent neurological deficits are observed in no more than 1% of patients in recent years (21, 23). In this study, surgical complications occurred in 9.3% of patients, and no permanent neurological deficits including hemiparesis, aphasia, dysphasia, and hemisensory deficit are observed. These may be because of the relatively limited sample size of the present study.

Factors including seizure duration, age at surgery, results of EEG, MRI results, history of status epilepticus, the extent of resection, and the present of secondary generalized tonic-clonic seizures, etc. have been reported as predictive factors of surgical outcomes in patients with TLE (24–28). However, the predictive value of these factors is limited in some special patient cohorts including patients with TLE secondary to FCD type IIIa because most of these previous studies included all TLE patients with diversiform etiology (29). In this study, we found that lesions on the right side independently predict seizure freedom in patients with TLE secondary to FCD type IIIa who had undergone ATL.

It has been reported that the side of lesions did not affect surgical outcomes in patients with mesial TLE (28). However, in patients with neocortical TLE, the side of lesions was reported to be associated with postoperative seizure outcomes (30). In this study, the TLE of all patients was secondary to FCD type IIIa, and we found that lesions on the right side independently predict postoperative seizure freedom. In these patients, HS and FCD type I in the adjacent temporal lobe could be observed during the histological evaluation (7). However, it is hard to diagnose the FCD type I in the temporal lobe preoperatively and intraoperatively because the reliable diagnostic tools are lacking, and the lesions of FCD type I in the temporal lobe are relatively diffuse (11, 31–33). When the surgical procedure was performed on the left side, the resection was more often incomplete because of the neighborhood of eloquent speech centers. And it has been demonstrated that a smaller extent of resection is significantly associated with unfavorable postoperative seizure outcomes (34, 35), which may explain the results of the present study. Recently, some studies have reported that some patients with left TLE had functional reorganization of language representation (36). In patients with left TLE, 20–33% of patients had right hemispheric or bilateral language representation (37–39). Thus, the performance of Wada test, functional magnetic resonance imaging and intraoperative language mapping to accurately locate the eloquent language centers may enable more extensive resection in the left TLE (40, 41).

## Limitations

The present study has some limitations. First, this is a single-center retrospective study, the inherent biases of the retrospective design cannot be ruled out. Second, the sample size of the present study is relatively small, large clinical samples in the future are helpful for establishing more definite conclusions. Third, the neuropsychological outcomes and postoperative quality of life are failed to evaluate in the present study, which is also important goals of surgical treatment for drug-resistant TLE. While these limitations were recognized, the present study still provides useful information about ATL for drug-resistant TLE due to FCD type IIIa.

## Conclusions

Data of the present study showed that ATL is an effective treatment for patients with drug-resistant TLE secondary to FCD type IIIa. Postoperative seizure freedom is more likely to be achieved in patients with lesions on the right side.

## Data Availability Statement

The raw data supporting the conclusions of this article will be made available by the authors, without undue reservation.

## Ethics Statement

The studies involving human participants were reviewed and approved by the ethics committee of Xiangya Hospital, Central South University. Written informed consent to participate in this study was provided by the participants' legal guardian/next of kin.

## Author Contributions

ZhiY contributed to the conception and design of the study. XH and DL collected the data. XH draft the manuscript. DL, ZhiY, JZ, and SL analyzed the results. All authors contributed to the article and approved the submitted version.

## Conflict of Interest

The authors declare that the research was conducted in the absence of any commercial or financial relationships that could be construed as a potential conflict of interest.
