# Supplementary material for: Side of Lesions Predicts Surgical Outcomes in Patients With Drug-Resistant Temporal Lobe Epilepsy Secondary to Focal Cortical Dysplasia Type IIIa
Source: Front Neurol. 2020 Dec 10;11:580221. doi: 10.3389/fneur.2020.580221 (PMC7758315; doi:10.3389/fneur.2020.580221)
Supplement: Supplementary file 1 [file Table_1.docx]

**Table S** intercorrelations between variables that were recruited into the multivariate analysis

| **Variables** | **Cramer's V** |
| --- | --- |
| **Side of lesions & Age at surgery** | 0.390 |
| **Side of lesions & Age at seizure onset** | 0.468 |
| **Side of lesions & IEDs** | 0.302 |
| **Age at surgery & Age at seizure onset** | 0.459 |
| **Age at surgery & IEDs** | 0.257 |
| **age at seizure onset & IEDs** | 0.314 |

IEDs, interictal epileptic discharges.
